# Self-harm and aggression in patients with anorexia nervosa

**DOI:** 10.1192/j.eurpsy.2021.1869

**Published:** 2021-08-13

**Authors:** N. Lebedeva, A. Parshukov, Y. Chebakova

**Affiliations:** 1 Clinical Psychology, Pirogov Russian National Research Medical University, Moscow, Russian Federation; 2 Psychology, Moscow Institute of Psychoanalysis, Moscow, Russian Federation

**Keywords:** anorexia nervosa, Aggression, self-harm

## Abstract

**Introduction:**

Anorexia nervosa (AN) is a complex condition with high comorbidity.

**Objectives:**

This study aims to verify whether patients with AN are more aggressive towards themselves than towards others; namely, we measure the levels of autoaggressive/aggressive ideation, negative emotions regarding self/others, as well as self-harm behavior.

**Methods:**

10 female patients with AN (2 of them also had bulimia nervosa) and 20 female participants of the control group were presented with Structured Interview, Rosenzweig Picture-Frustration Test (P‐F), Thomas-Kilmann Conflict Mode Instrument, Buss-Durkee Hostility Inventory, I-structural Test of Ammon, Boyko Communication Aggression Inventory, Boyko Self-directed Emotion Accumulation Inventory (BSEAI). Mann-Whitney U-test and Pearson’s correlation coefficient were used.

**Results:**

**Table:** 

Table 1		
**Structured interview indicator**	**Control group**	**AN patients**
Suicide attempts	10%	60%
Self-harm behavior	35%	60%
Piercing/tattoos	20%	50%
Depression (self-report)	40%	90%

There were no significant differences in aggression levels between groups. However, patients with AN showed less extrapunitive reactions: blaming others, requiring others to resolve the situation (P-F, р=0.013) and more intropunitive reactions: self-blame, feeling responsible/guilty for the situation (P-F, р=0.031). AN patients had more self-directed negative emotions and impulses (BSEAI, р=0.01), more self-harm behavior (see table 1). There were no correlations between autoaggression and aggression scales in the control group, but there were 9 correlations between them in the AN group (p<0.05, r>0.76).

**Conclusions:**

Patients with AN are more inclined to self-blame, negative ideas about themselves, self-harm behavior, but have the same aggression level as the control group. The interconnection of aggression and autoaggression is different in patients with anorexia nervosa compared to the control group.

**Disclosure:**

No significant relationships.

